# Correction to: A Genome-Wide Screen for Genes Affecting Spontaneous Direct-Repeat Recombination in *Saccharomyces cerevisiae*

**DOI:** 10.1093/g3journal/jkac181

**Published:** 2022-08-11

This is a correction to: Daniele Novarina, Ridhdhi Desai, Jessica A Vaisica, Jiongwen Ou, Mohammed Bellaoui, Grant W Brown, Michael Chang, A Genome-Wide Screen for Genes Affecting Spontaneous Direct-Repeat Recombination in *Saccharomyces cerevisiae*, *G3 Genes|Genomes|Genetics*, Volume 10, Issue 6, 1 June 2020, Pages 1853–1867, https://doi.org/10.1534/g3.120.401137

In the originally published version of this manuscript a mistake was made in making Table S3 that caused the pinning data for several hundred strains to be assigned to an incorrect genotype. The authors have corrected the problem and the main conclusions of the paper still hold true.

Corrected supplemental tables have been posted at figshare: https://doi.org/10.25387/g3.11830833.

The changes are:

1. The raw and filtered data of the high-throughput replica pinning screen have been corrected in Tables S3 and S4, respectively. Table S6 (enriched GO terms) has also been updated.
2. A new Table 1 with 64 genes (instead of 75) identified as hyper-rec from the pinning assay. *ELG1*, *ALE1*, *NFI1*, *EFT1*, *PNS1*, *ARP8*, *DCS2*, *PET123*, *RGS2*, *SPR1*, and *ULS1* were removed because they contain an extra mutation of *msh3*. *CSM1* and *NUP170* disappear from the top of the list (both are false negatives since it was found that they do have increased rates of recombination; Table S5), and *MGS1*, *YDL162C*, and *MPRL9* appear. *MGS1* and *YDL162C* were also identified in the patch assay screen.
3. A new Table 2 of hypo-rec screen hits from the pinning assay.
4. Figures 2D and 3A have been updated. The new hypergeometric *P* value for the overlap between the patch and pinning screens shown in Figure 3A is 8.2 × 10^-23^.

**Table 1.** Hyper-recombination genes from the patch assay and pinning assay screens.

Patch Assay				Pinning Assay Hyper-Rec			
Gene name	Mean recombination rate^a^	Standard deviation	p-value^b^	Gene name	Recombinant colonies (%)	Gene name	Recombinant colonies (%)
WT	1.14E-05	2.84E-06		*MSH2*	100	*YGL159W*	90
*TSA1*	1.23E-04	3.64E-05	7.76E-05	*RAD27*	100	*YJL043W*	90
*VMA11*	1.19E-04	7.62E-06	1.27E-08	*RRM3*	100	*YLR279W*	90
*RAD27*	9.39E-05	2.59E-05	1.26E-04	*SGS1*	100	*YOR082C*	90
*RMI1*	7.50E-05	6.85E-06	2.65E-07	*TSA1*	100	*BNA1*	88
*TOP3*	6.15E-05	3.80E-06	1.13E-07	*YDL162C*	100	*COX7*	88
*SKN7*	5.80E-05	6.85E-06	2.20E-06	*DST1*	98	*DDC1*	88
*APN1*	5.75E-05	2.97E-05	3.79E-03	*RNH202*	98	*FUS2*	88
*ELG1*	5.09E-05	1.30E-05	1.73E-04	*RNH203*	98	*HST3*	88
*MLH1*	4.86E-05	1.15E-05	3.43E-05	*MLH1*	96	*IRC6*	88
*RNH203*	4.68E-05	6.79E-06	1.31E-05	*MRPL9*	96	*MFT1*	88
*YLR235C*	4.52E-05	2.57E-06	6.11E-07	*PMS1*	96	*MNT2*	88
*TOF1*	4.39E-05	1.40E-05	9.45E-04	*APN1*	94	*MRPL51*	88
*YAP1*	4.22E-05	5.04E-06	8.67E-06	*YGR117C*	94	*NIT3*	88
*RNH202*	3.96E-05	1.38E-05	1.96E-03	*YML020W*	94	*PCL10*	88
*RNH201*	3.86E-05	6.08E-06	1.91E-06	*MME1*	94	*PHM8*	88
*SGS1*	3.75E-05	1.42E-05	2.25E-03	*YOR072W*	94	*RBS1*	88
*YDL162C*	3.34E-05	9.73E-06	1.38E-03	*MGS1*	94	*REC107*	88
*PMS1*	3.33E-05	1.28E-05	3.46E-03	*DIA2*	92	*REC114*	88
*HYR1*	3.16E-05	1.74E-05	1.85E-02	*MDM1*	92	*RPP1B*	88
*MGS1*	3.10E-05	3.83E-06	6.14E-05	*MSN4*	92	*SCO1*	88
*MSH2*	3.09E-05	1.34E-06	1.55E-06	*RHO2*	92	*SCW11*	88
*DST1*	3.07E-05	6.56E-06	1.15E-04	*RMI1*	92	*TOM5*	88
*YER188W*	2.99E-05	1.27E-05	9.90E-03	*SAC3*	92	*YDL009C*	88
*CSM3*	2.64E-05	3.65E-06	2.78E-04	*YDR230W*	92	*YDL172C*	88
*HTA2*	2.60E-05	6.24E-06	1.87E-03	*YLR235C*	92	*YEL020C*	88
*RAD4*	2.35E-05	2.46E-06	1.73E-03	*YNL122C*	92	*YGL042C*	88
*MSH6*	2.34E-05	1.02E-05	1.68E-02	*YTA7*	92	*YJR124C*	88
*RAD6*	2.22E-05	7.25E-06	7.23E-03	*FSH1*	92	*YKL091C*	88
*RRM3*	2.16E-05	8.30E-06	1.54E-02	*GET3*	92	*YKL162C*	88
*CHL4*	2.14E-05	5.86E-06	9.36E-03	*KGD2*	92		
*THP2*	1.94E-05	2.95E-06	2.52E-03	*MID2*	92		
*DFG16*	1.80E-05	4.48E-06	2.44E-02	*POL32*	92		
*ABZ2*	1.66E-05	3.93E-06	4.25E-02	*RNH201*	90		

a Recombination rate from Table S2

b p-values from one-sided Student's *t*-test

**Table 2.** Hypo-recombination genes from the pinning assay screen.

Pinning Assay Hypo-Rec							
Gene name	Recombinant colonies (%)	Gene name	Recombinant colonies (%)	Gene name	Recombinant colonies (%)	Gene name	Recombinant colonies (%)
*YCL021W-A*	0.0	*SIP3*	17.2	*PHO85*	27.1	*AIM39*	31.3
*YEL045C*	0.0	*BEM1*	18.8	*PRM4*	27.1	*CIK1*	31.3
*GLY1*	0.0	*BUB3*	18.8	*RAD57*	27.1	*HOL1*	31.3
*HIS5*	0.0	*OPI3*	18.8	*RIM1*	27.1	*MET22*	31.3
*RAD52*	2.1	*DCC1*	18.9	*UBP15*	27.1	*OSH1*	31.3
*GCN4*	2.9	*ARG7*	19.1	*VMA21*	27.1	*RNR4*	31.3
*CYS4*	3.1	*URA4*	19.6	*YBR075W*	27.1	*RPN4*	31.3
*POS5*	3.1	*OPY2*	20.0	*AAT2*	27.5	*RPS18B*	31.3
*KCC4*	4.2	*YGL218W*	20.0	*RAD50*	27.8	*SWI4*	31.3
*LEU3*	4.2	*DAL81*	20.9	*ARG2*	28.1	*TSL1*	31.3
*ATP15*	4.8	*TFB5*	21.3	*RNR1*	28.2	*VPS60*	31.3
*YPR099C*	4.9	*RPL22A*	21.6	*RPS8a*	28.2	*VTH1*	31.3
*ACO2*	6.4	*RSM7*	21.7	*YKR023W*	28.6	*YKE2*	31.3
*MDM20*	6.4	*CCR4*	22.2	*ATP1*	29.2	*YNR040W*	31.3
*MDM10*	6.9	*LOC1*	22.2	*FIT2*	29.2	*NUP84*	31.6
*NPL3*	7.1	*AHC1*	22.9	*HSP42*	29.2	*ATG4*	31.7
*HIS7*	7.7	*CIN1*	22.9	*MRPS16*	29.2	*URA2*	31.7
*FUN12*	8.3	*VRP1*	22.9	*RAD54*	29.2	*RNQ1*	31.8
*BDF1*	11.1	*YEL014C*	22.9	*RAD55*	29.2	*THP1*	31.8
*TPD3*	12.5	*CDC40*	23.1	*SNO1*	29.2	*BUD20*	32.1
*SWI6*	12.8	*MDM34*	23.4	*SPE2*	29.2	*RPS16A*	32.6
*URA1*	13.2	*YGL188C-A*	24.0	*SPT21*	29.2		
*YGR272C*	13.2	*YCR007C*	24.3	*TCD1*	29.2		
*BUD19*	13.3	*KNH1*	25.0	*TPM1*	29.2		
*UGO1*	13.3	*SHE4*	25.0	*YDR157W*	29.2		
*ZWF1*	14.6	*SNF6*	25.0	*YFR012W-A*	29.2		
*SWI3*	14.8	*YJR011C*	25.0	*SFB3*	29.6		
*YOR302W*	15.0	*AGP1*	25.7	*YME1*	29.6		
*YCL022C*	15.2	*ACM1*	25.9	*NGG1*	30.3		
*ACE2*	15.6	*YCL023C*	26.0	*POP2*	30.4		
*MOT2*	15.8	*FEN2*	26.7	*ATP11*	30.8		
*YDR444W*	15.8	*BUB1*	26.8	*RPL37B*	31.0		
*SLX5*	16.7	*CCW12*	27.1	*HFI1*	31.0		
*SLX8*	16.7	*HST4*	27.1	*YML013C-A*	31.1		

**Figure 2.**
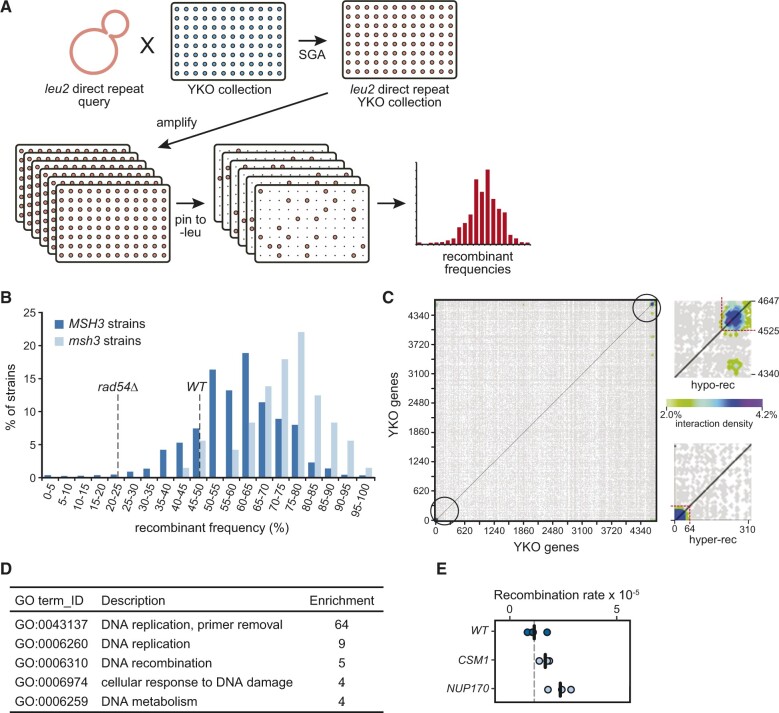
A high-throughput replica-pinning screen for genes controlling direct-repeat recombination. (A) Schematic representation of the screen based on high-throughput replica-pinning. The leu2 direct-repeat recombination cassette was introduced into the yeast deletion collection as in Figure 1B. The resulting strains were amplified by parallel high-throughput replica pinning and subsequently replica-pinned to media lacking leucine to select for recombination events. Recombinant frequencies were calculated for each strain of the YKO collection. (B) Recombinant frequency distribution for the YKO collection (*MSH3* strains) and for the *msh3* strains in the collection. Recombinant frequencies for a wild-type and for a recombination-defective *rad54Δ* strain derived from a pilot experiment are indicated by the dashed lines. (C) Interaction densities determined by CLIK analysis are plotted as a two-dimensional heatmap. The cutoffs established by CLIK analysis for hyper-recombination (hyper-rec) and recombination-defective (hypo-rec) genes are shown in the insets. (D) The statistically supported GO terms enriched in the hits from the pinning assay screen are shown, with the enrichment for each term. (E) Recombination rates from fluctuation tests of *csm1**Δ* and *nup170**Δ* are plotted. Each data point is from an independent fluctuation test, with n = 3 for each strain. The vertical bars indicate the mean recombination rate for each strain and the wild-type data from Figure 1D are plotted for comparison.

**Figure 3.**
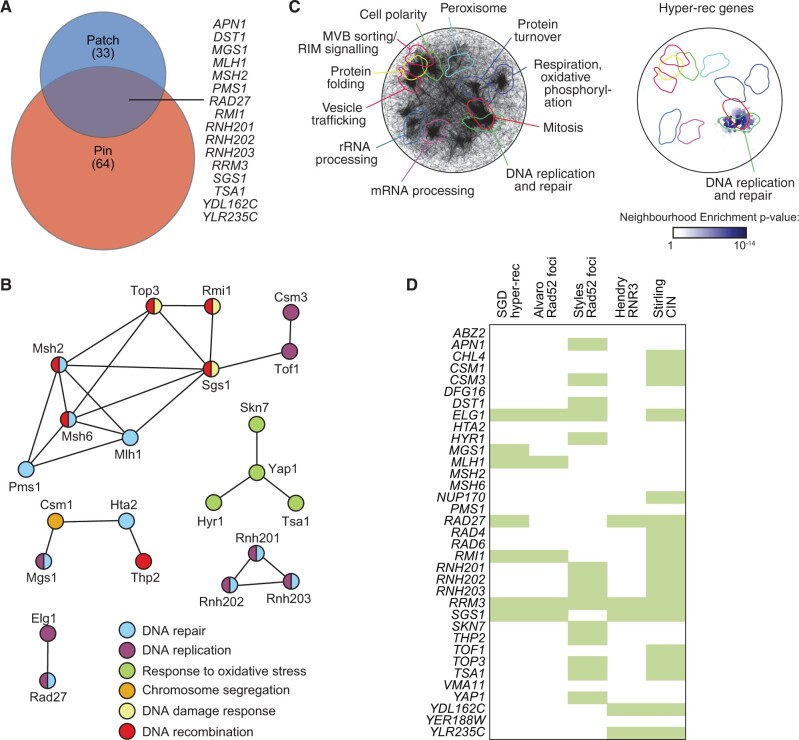
Functional analysis of validated hyper-rec genes. (A) The overlap of the hyper-rec genes for the two screens is plotted as a Venn diagram. The 16 genes identified in both screens are indicated. (B) A protein-protein interaction network for the proteins encoded by the 35 validated hyper-rec genes is shown. Nodes represent the proteins, and are colored to indicate function. Edges indicate a physical interaction as annotated in the GeneMania database. (C) Spatial analysis of functional enrichment. On the left, the yeast genetic interaction similarity network is annotated with GO biological process terms to identify major functional domains (Costanzo *et al.* 2016). 11 of the 17 domains are labeled and delineated by colored outlines. On the right, the network is annotated with the 35 validated hyper-rec genes. The overlay indicates the functional domains annotated on the left. Only nodes with statistically supported enrichments (SAFE score > 0.08, *P* < 0.05) are colored. (D) The 35 validated hyper-rec genes are compared with existing *Saccharomyces* Genome Database annotations and genome instability datasets that measured Rad52 focus formation (Alvaro *et al.*, 2007; Styles *et al.*, 2016), *RNR3* induction (Hendry *et al.*, 2015), or chromosome instability (CIN; (Stirling *et al.*, 2011)). A green bar indicates that the gene has the given annotation or was detected in the indicated screen.

These details have been corrected only in this correction notice to preserve the published version of record. 

